# The Effect of *Oliveria Decumbens* and *Pelargonium Graveolens* on Healing of Infected Skin Wounds in Mice

**Published:** 2016-09

**Authors:** Mohaddese Mahboubi, Mohammad Mehdi Feizabadi, Tahereh Khamechian, Nastaran Kazempour, Mohsen Razavi Zadeh, Farhang Sasani, Mohsen Bekhradi

**Affiliations:** 1 Microbiology Department, Medicinal Plant Research Center of Barij, Kashan, Iran;; 2 Department of Microbiology, School of Medicine, Tehran University of Medical Science, Tehran, Iran;; 3 Department of Pathology, Faculty of Medicine, Kashan University of Medical Sciences, Kashan, Iran;; 4 Department of Gastroenterology, Beheshti Hospital, Kashan University of Medical Sciences, Kashan, Iran;; 5 Department of Pathology, Faculty of Veterinary Medicine, Tehran University, Tehran, Iran;; 6 Formulation Department, Medicinal Plant Research Center of Barij, Kashan, Iran

**Keywords:** Oliveria decumbens, Pelargonium graveolens, Healing, Infected wound, Mouse

## Abstract

**BACKGROUND:**

*Staphylococcus aureus* is one of the most causative organisms in the skin wound infections. Development of resistant* S. aureus* to current treatments in individuals with low immunity is a global concern. The aim of this study was to evaluate the efficacy of herbal formulation against skin wound infection**.**

**METHODS:**

The efficacy of herbal formulation containing *Oliveria decumbens* and *Pelargonium graveolens* essential oils was evaluated in comparison to mupirocin against Methicillin Resistant *S. aureus* (MRSA) related skin wound infection in mice animal model.

**RESULTS:**

The herbal cream and mupirocin decreased the log CFU by 2.5±0.26 and 2.46±0.32, respectively, while the log CFU of *S. aureus* from wound skin were 5.9±0.26 and 5.65±0.23 for placebo and control groups, respectively. Moreover, the histological examinations showed that this cream improved the wound healing and increased the collagen deposition and wound contraction.

**CONCLUSION:**

This natural new formulation with *O. decumbens* and *P. graveolens* essential oils could be recommended as a new candidate for wound healing.

## INTRODUCTION

The common bacterial diseases of the skin are impetigo, pitted keratolysis, erysipelas, cellulitis, folliculitis, furunculosis and carbuncle. These infections involve the superficial epidermis, dermis, and subcutaneous tissue, superficial and deep follicles. The main causative organisms are *Staphylococcus aureus*, *Streptococcus pyogenes*, *Micrococcus sedentarius*, *Dermatophilus congolensis*, and *Pseudomonas aeruginosa*. However, *Staphylococi* particularly *S. aureus* is the likely common cause of boils, pustules, carbuncles and post operative infections. The immune-compromised individuals, elderly, children and hospitalized patients are very susceptible to these bacterial infections. 

Mupirocin is the current topical therapy for Staphylococcal infection. The prevalence of mupirocin resistance in methicilin resistant (MRSA) and vancomycin resistant *S. aureus* have previously been reported.^1^^,^^2^ Development of *S. aureus* resistance to current treatments in individuals with low immunity is a global concern. This has been led to seek for finding new compounds mainly among the naturals with the goal to discover new antimicrobial agents. The essential oils are secondary metabolites of plants that have multifunctional effects including wound healing effects, immune-enhancing and antibacterial agents. Many essential oils are known as antimicrobial agents and are used for healing of infected skin wounds.^3^^-^^11^

Den oil is extracted from *Oliveria decumbens *aerial part at full flowering stage (Umbelifera family). It is used as antiseptic agent in traditional medicine and is known as a broad spectrum antimicrobial agent due to its strong antibacterial activity against *S. aureus*, *Escherichia coli*, *P. aeruginosa,* *Aspergillus niger*, and *Candida albicans*.^6^^,^^9^^,^^10^ The antimicrobial activity of den oil is due to thymol and carvacrol as its main components.^3^ Geranium oil is the colorless or green blue liquid oil from *Pelargonium graveolens* leaves that is famous due to its rose like odor and its wound healing effects. Citronellol and geraniol are the dominant compounds of geranium oil.^7^ It has the valuable antimicrobial activity against *C. albicans*,^7^ *P. aeruginosa*,^5^ *S. aureus*,^8^ *E. coli*, *A. niger*.^9^ In this study, we developed a new herbal formulation containing the geranium and den oils against MRSA related infected skin wound in mice animal model and evaluated its efficacy in comparision with mupirocin as current treatment.

## MATERIALS AND METHODS

*O. decumbens* and *P. graveolens* aerial parts were collected in May 2013 from the Kazeron area in the Fars province and Research Farm of Barij Essence in Kashan, respectively. The hydro-distillation method was used to extract the essential oils by Clevenger-type apparatus for 6 h. The oils were analyzed using GC-FID and GC-MS. The GC-FID and GC-MS apparatus were conducted on an HP 6890 GC system coupled with 5973 network mass selective detectors with a capillary column of HP-5MS (30 m × 0.25 mm, film thickness 0.25 µm). 

The oven temperature program was initiated at 60°C, held for 1 min, then raised to 245°C at a rate of 3°C/min held for 10 min. Helium was used as the carrier gas at a flow rate 1.5 ml/min. The detector and injector temperatures were 250 and 230°C, respectively. Quantization of major constituents of the oils was performed by the area normalization method. The compounds of the oil were identified by comparison of their retention indices (RI), mass spectra fragmentation with those on the stored Wiley 7n.1 mass computer library.^12^ The herbal formulation was prepared from Barij Essence Pharmaceutical Company. 

In detail, oil phase and aqueous phase of cream was heated to 73ºC, separately. Aqueous phase was added to oil phase; until the emulsion was formed. Afterward, the emulsion was cooled until 45ºC and the oils were inserted and mixed. The placebo cream (Barij Essence Pharmaceutical Company) was including all of the components excepting the active ingredient. Mupirocin ointment was purchased from Pars Daru, Tehran, Iran and was used as positive control in this study.

Male mice (Balb/C), average weighing 23 grams were obtained from Razi Inisitute, Hesarak, Karaj, Iran. The animals have free access to food and water *ad libitum*. The experimental subjects were kept in a single holding room and housed in a constant temperature of 21±2^o^C, humidity of 55±5% and under 12-h light/dark cycle. All experiments were in accordance with the UK Animals Scientific Procedures Act 1986 (86/609/EEC). The mice were divided into four groups. Each group containing 30 animals were spread in three cages and were housed in polycarbonate cages. 

Animal infection experiments were performed at the Microbiology Department, Animal House of Research Center of Barij, Kashan, Iran. The established skin suture wound model was carried out as described by Gisby and Bryant.^13^ Methicilin Resistant *S. aureus* (MRSA) was isolated from the skin wound of an infected patient at Tehran University’s hospital and was confirmed by biochemical tests and culturing on ChromAgar media (Himedia, India). MRSA was inoculated in nutrient broth containing 0.2% yeast extract and incubated for one night. Sterile silk sutures number 3/0 (Supasil, Karaj, Iran) were cut into 20-cm length and soaked in undiluted overnight broth culture for 30 minute. 

Infected sutures were removed aseptically and put on sterile filter in plate and stored at the 4ºC until the surgeries were performed on animals. Five samples of different sutures were cut into 1-cm and CFU were determined. Anesthesia was induced by peritoneal injection of diazepam (Khemidaru, Iran) at 1.43 mg/kg, along with 10% ketamine (alfasan, Woerden-Holland) at 13 mg/kg. The furs of the back of mice were stripped and the skin of back was swabbed with 70% ethanol. One centimeter of infected silk suture was inserted under the clipped skin and knotted the suture. One incision was made along with the suture. One wound was created per animal. The wound was closed with an adhesive temporary skin closure and after 4 h, treatment was initiated. 

Mupirocin ointment (I), natural herbal cream (II) and placebo (III) were applied (0.1 ml volume) on the wounds and was spread over the area. One group was left as untreated group as infection control (IV). The second application was made after 6 h and later treatments were continued three times daily for a future 7 days. Sixteen to twenty hours after the last topical application, animals were sacrificed by CO_2_. Furs around the wounds were clipped if it was necessary. The skin was swabbed by ethanol. 

Area (1-2 cm) of skin including the wound was excised and homogenized in 1 ml of yeast extract. The homogenates yeast extract were serially diluted and enumerated. The Bacterial counts were expressed as means log CFU±standard deviations (SD). In order to characterize the histopathology of the skin in treated and untreated animals, the biopsy specimens were taken from the wound infected skin and immediately put in formalin solution (10%). The formalin-fixed biopsy specimens were embedded in paraffin, were sliced and stained by hematoxylin and Eosin (H&E). The samples were observed blindly by histopathology’s investigator and the treatment response were reported.

Statistical analysis of the log_10_-transformed data was performed to ensure variance homogeneity and normality. Thus, an analysis of variance (generalized linear models) was applied, followed by four predefined pair wise treatment comparisons adjusted for multiplicity by the Bonferroni method, yielding a statistical ranking. Furthermore, to ensure robustness in the analysis performed, the nonparametric Kruskal-Wallis approach was used. The method showed no conclusive dissimilarities to the generalized linear models approach. All testing was performed on an overall 5% significance level, meaning that P values less than 0.05 were considered as statistically significant difference (*P*<0.05). 

## RESULTS

The main components of *O. decumbens* essential oil were thymol (50.1%), *γ *-terpinene (20.7%), and p-cymene (17.6%) while* β*-citronellol (39.3%) and geraniol (23.6%) were the major compounds in *P.* *graveolens *oil. The results of log CFU of *S. aureus* in different groups (after treatment) showed that treatment with herbal cream and mupirocin significantly decreased the log CFU by 2.5±0.26 and 2.46±0.32, respectively, while the log CFU of *S. aureus* from wound skin were 5.9±0.26 and 5.65±0.23 for placebo and control groups, respectively. There was no significant difference between herbal cream and mupirocin and also between control and placebo groups (*p*>0.05), while there was significant difference between herbal cream and mupirocin with control and placebo groups (*p*<0.05). The herbal cream had the same efficacy like mupirocin in reducing the CFU of *S. aureus*. 

The histopathological analysis of the skin in different groups showed that control group had the thin epidermis and the central part of the epidermis was very thin (scars) with congestion and low fibroblast. The dermis had infiltration of inflammatory cells and intense accumulation of inflammatory cells (inflammation phase). Placebo group had almost normal epidermis but the dermis had severe infiltration of inflammatory cells, necrosis and abscess formation (inflammation phase). 

**Fig. 1:**
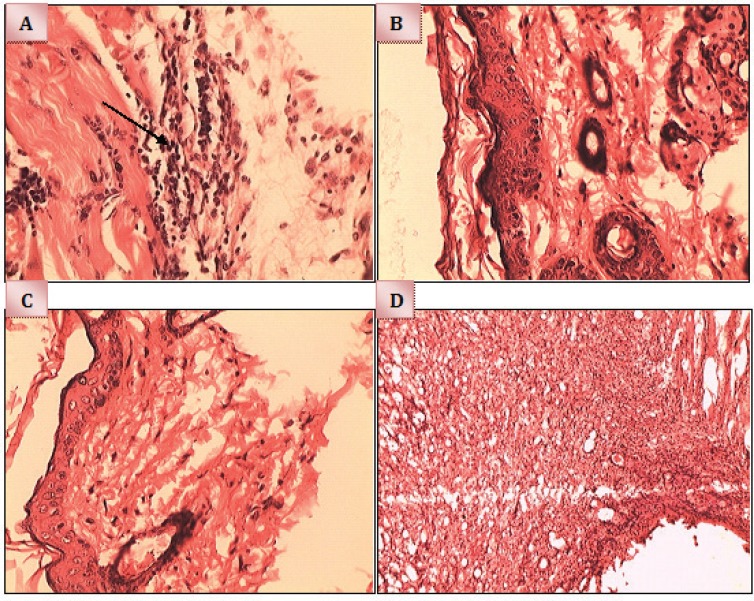
Microscopic evaluation of Hematoxylin & Eosin stained sections (Ma ×40). A) Control, B) Herbal cream, C) Mupirocin, D) Placebo (Control).

In Mupirocin group, the thickness of the epidermis was the same in almost all areas. Mild infiltration of inflammatory cells in the dermis with low collagen fibers, low congestion and edema with low fibroblasts were seen (Overlapping of inflammation and proliferation phases). In herbal cream group, epidermis and the dermis was normal, in very limited area of dermis, infiltration of very mild inflammatory cell were noted. Collagen deposition and granulation of tissue were visible (proliferation phase) (Figure 1).

## DISCUSSION

The results of our study showed that the herbal cream had the good efficacy in preventing the skin from bacterial infections and in tissue repairing and these potencies were comparable to mupirocin. The good efficacy of this herbal cream in controlling of infection was related to its essential oils. Geranium oil was one of the main components of this formulation. The antibacterial activity of geranium essential oil against *S. aureus*^14^^-^^16^ has previously been confirmed. 

In one study, among the patchouli, tea tree, geranium and lavender essential oils; geranium oil vapors showed the greatest-anti-bacterial effects against MRSA.^16^ Also an essential oil blend containing lemongrass and geranium oils reduced the MRSA growth on seeded plates by 38% after 20 h exposure.^15^ In other study, geranium oil had strong activity against clinical *S.* *aureus* isolates-including multidrug resistant strains, exhibiting the MIC values of 0.25-2.50 μl/ml.^14^

Other active component of herbal cream was den essential oil, the anti-staphylococcal activity of den essential oil^3^^,^^10^ were reported before. The antimicrobial properties of oils inhibit the growth of bacteria from developing on the wounds. Therefore, the potency of herbal cream in control of *S.* *aureus* infections was related to the strong anti-staphylococcal activity of both essential oils. The efficacy of cream to repair of skin tissue may be correlated to geranium and den oil. 

Wound healing is classically divided into hemostasis, inflammation, proliferation, and remodeling. One of the main phases in wound healing is collagen deposition and wound contraction that shows the skin is in the proliferation phase of wound healing process. The results of our histopathological assessments showed that herbal cream increased collagen deposition and wound contraction; therefore, it increases the proliferation of cell skin. It is reported geranium oil has repairing effects and has been discussed as being useful in healing wounds and abscesses^17^^,^^18^ and is suitable for any skin type such as oily or congested skin, eczema, and dermatitis and may also be one of the best oils for diverse dermatological problems.^4^^,^^19^

It also reduces the inflammation while still being stimulating, regenerating skin cells. Previously, it was reported that the intraperitoneally administration of geranium oil suppressed the leukocyte recruitment resulted from inhibition of neutrophil accumulation into the peritoneal cavity,^20^ and coetaneous application of geranium oil suppress the cellular inflammation induced by curdlan dose-dependently and effectively inhibited neutrophil accumulation in vivo.^21^

There is no information about the effect of den essential oil on wound healing, but thymol as the main component of this essential oil has been indicated as potential agent in the treatment of inflammation and wound healing and improved wound retraction rates, granulation reaction, better collagenization density and arrangement during the process of wound healing.^22^ Our findings demonstrated that presence of *O. decumbens* and P. graveolens oils in herbal cream can have antimicrobial effect and healing properties and these herbals can be recommended in treatment of infected skin wounds. More clinical and toxicological studies are required to demonstrate its efficacy in patients.
